# Nebulin interactions with actin and tropomyosin are altered by disease-causing mutations

**DOI:** 10.1186/2044-5040-4-15

**Published:** 2014-08-01

**Authors:** Minttu Marttila, Mubashir Hanif, Elina Lemola, Kristen J Nowak, Jenni Laitila, Mikaela Grönholm, Carina Wallgren-Pettersson, Katarina Pelin

**Affiliations:** 1 The Folkhälsan Institute of Genetics, Biomedicum Helsinki, Helsinki, Finland; 2 Department of Medical Genetics, Haartman Institute, University of Helsinki, Helsinki, Finland; 3 Molecular Neurogenetics Laboratory, Centre for Medical Research, University of Western Australia, Nedlands, Australia; 4 Department of Biosciences, Division of Biochemistry and Biotechnology, University of Helsinki, Helsinki, Finland; 5 Department of Biosciences, Division of Genetics, University of Helsinki, Helsinki, Finland

**Keywords:** Nemaline (rod) myopathy, Congenital myopathy, Nebulin, Actin, Tropomyosin and protein binding

## Abstract

**Background:**

Nemaline myopathy (NM) is a rare genetic muscle disorder, but one of the most common among the congenital myopathies. NM is caused by mutations in at least nine genes: Nebulin (*NEB*), α-actin (*ACTA1*), α-tropomyosin (*TPM3*), β-tropomyosin (*TPM2*), troponin T (*TNNT1*), cofilin-2 (*CFL2*), Kelch repeat and BTB (POZ) domain-containing 13 (*KBTBD13*), and Kelch-like family members 40 and 41 (*KLHL40* and *KLHL41*). Nebulin is a giant (600 to 900 kDa) filamentous protein constituting part of the skeletal muscle thin filament. Around 90% of the primary structure of nebulin is composed of approximately 35-residue α-helical domains, which form super repeats that bind actin with high affinity. Each super repeat has been proposed to harbor one tropomyosin-binding site.

**Methods:**

We produced four wild-type (WT) nebulin super repeats (S9, S14, S18, and S22), 283 to 347 amino acids long, and five corresponding repeats with a patient mutation included: three missense mutations (p.Glu2431Lys, p.Ser6366Ile, and p.Thr7382Pro) and two in-frame deletions (p.Arg2478_Asp2512del and p.Val3924_Asn3929del). We performed F-actin and tropomyosin-binding experiments for the nebulin super repeats, using co-sedimentation and GST (glutathione-S-transferase) pull-down assays. We also used the GST pull-down assay to test the affinity of WT nebulin super repeats for WT α- and β–tropomyosin, and for β-tropomyosin with six patient mutations: p.Lys7del, p.Glu41Lys, p.Lys49del, p.Glu117Lys, p.Glu139del and p.Gln147Pro.

**Results:**

WT nebulin was shown to interact with actin and tropomyosin. Both the nebulin super repeats containing the p.Glu2431Lys mutation and nebulin super repeats lacking exon 55 (p.Arg2478_Asp2512del) showed weak affinity for F-actin compared with WT fragments. Super repeats containing the p.Ser6366Ile mutation showed strong affinity for actin. When tested for tropomyosin affinity, super repeats containing the p.Glu2431Lys mutation showed stronger binding than WT proteins to tropomyosin, and the super repeat containing the p.Thr7382Pro mutation showed weaker binding than WT proteins to tropomyosin. Super repeats containing the deletion p.Val3924_Asn3929del showed similar affinity for actin and tropomyosin as that seen with WT super repeats. Of the tropomyosin mutations, only p.Glu41Lys showed weaker affinity for nebulin (super repeat 18).

**Conclusions:**

We demonstrate for the first time the existence of direct tropomyosin-nebulin interactions *in vitro*, and show that nebulin interactions with actin and tropomyosin are altered by disease-causing mutations in nebulin and tropomyosin.

## Background

Nemaline myopathy (NM) is a neuromuscular disorder characterized by muscle dysfunction and the presence of nemaline bodies (rods) in the muscle fibers. The rods are composed of thin filament and Z-disk proteins [1-4]. To date, nine different causative genes have been identified for NM: nebulin (*NEB*), skeletal muscle α-actin (*ACTA1*), slow α-tropomyosin (*TPM3*), β-tropomyosin (*TPM2*), slow troponin T (*TNNT*), cofilin-2 (*CFL2*), Kelch repeat and BTB (POZ) domain-containing 13 (*KBTBD13*; a member of the BTB/Kelch protein family), and Kelch-like family members 40 and 41 (*KLHL40, KLHL41*) [5-13]. Mutations in *NEB* and *ACTA1* are the most common causes of NM. In addition to NM, mutations in *NEB* cause distal nebulin myopathy with no or almost no nemaline bodies, a condition so far described only in Finland [14], distal myopathy with nemaline bodies (distal nemaline myopathy) [15], and rare cases of core-rod myopathy [16].

A rapidly growing number of mutations in the human *NEB* gene have been identified as a common cause of NM. These *NEB* mutations include frameshifts, premature stop codons, splice-site mutations, large in-frame deletions, and missense mutations [6,17-19]. The mutations cause both mild and severe forms of NM, although the typical congenital form appears to be the most common, which usually results only in slowly progressive disease [1,2]. Homozygous missense mutations in *NEB* have been found to cause distal nebulin myopathy [14], and *NEB* compound heterozygous mutations may result in core-rod myopathy [16].

Nebulin is a giant (600 to 900 kDa), thin-filament, actin-binding protein, and the gene comprises a total of 183 exons, of which at least 17 are alternatively spliced, producing hundreds of different *NEB* isoforms [20]. A major stretch of nebulin consists of repetitive modules, 30 to 35 amino acids (aa) long, called simple repeats [21]. Most of these simple repeats are arranged into seven-module super repeats. Each simple repeat has a predicted α-helical secondary structure, and an SDXXYK motif that serves as an actin-binding site [22,23]. A second motif, WLKGIGW, is present once in each super repeat, and is thought to serve as a tropomyosin-binding site [21]. The longest isoforms of nebulin bind as many as 239 actin monomers, and are thought to act as molecular rulers, defining thin-filament lengths, especially specifying minimum lengths of the filaments to optimize thin/-thick filament overlap and force production [24-26]. Apart from thin-filament regulation, the structural roles of nebulin extend to maintaining intermyofibrillar connectivity through interaction with desmin [27] and setting physiological Z-disk widths [24,28]. Knockout *Neb*^ΔExon55^ mice show impaired regulation of contraction, which appears as marked changes in cross-bridge cycling kinetics and a reduction in the calcium sensitivity of force generation [29].

Biochemical studies have shown that isolated nebulin super repeats bind actin with high affinity [30]. Furthermore, a nebulette repeat 167 aa long, consisting of five nebulin-like repeats approximately 35 aa long, was shown to interact with actin, tropomyosin, and the troponin complex [31]. This fragment shows the highest homology to nebulin C-terminal simple repeats outside the super repeat region of nebulin. During muscle contraction, tropomyosin moves between different binding sites on the actin filament, allowing actin-myosin interactions [32]. It also appears that nebulin has several binding sites on actin, suggesting that nebulin acts in concert with tropomyosin during muscle contraction [32].

**Figure 1:**
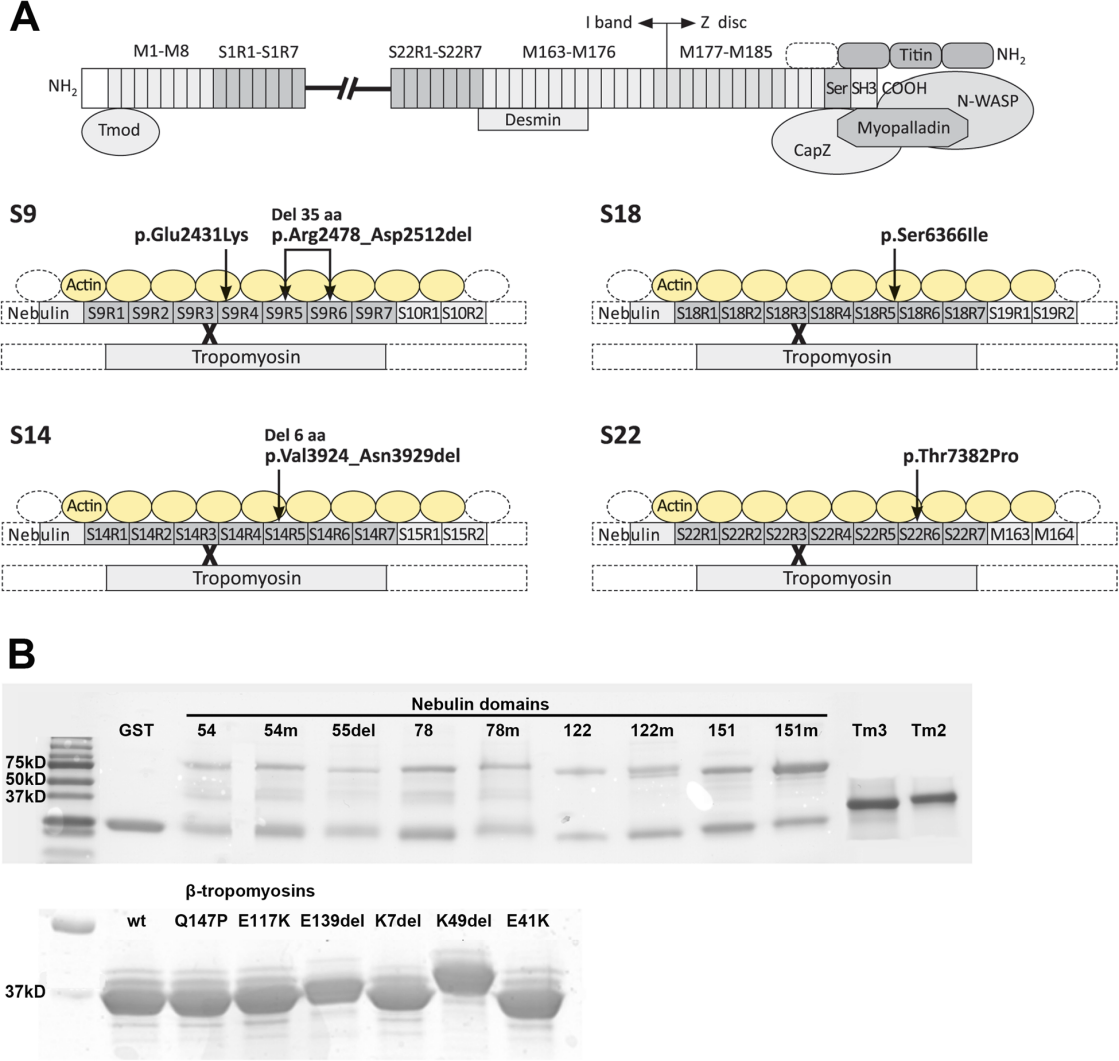
**Nebulin and β-tropomyosin mutations studied. (A)** The nebulin protein structure and the location of the mutations. The upper part of the figure shows a schematic presentation of the nebulin protein structure and its known protein interaction partners. The lower part of the figure shows a detailed view of the super repeats included in the study (S9, S14, S18, and S22), and the location of the mutations (arrows) and tropomyosin-binding sites (X) in the super repeats. **(B)** Purified GST (glutathione-S-transferase)-nebulin and tropomyosin. GST-nebulin domains were produced in the *Escherichia coli* strain BL21 (upper panel), and the α-tropomyosins (Tm3) and β-tropomyosins (Tm2) in insect cells (lower panel). The proteins were purified, run in a SDS-PAGE gel, and stained with Coomassie Blue. The β-tropomyosin mutations p.K49del and p.E139del cause altered protein conformation and thus slower migration in the SDS-PAGE gel [35]. Nomenclature of the mutations in relation to other figures: p.Glu2431Lys = ex54m, p.Arg2478_Asp2512del = ex55del, p.Val3924_Asn3929del = ex78m, p.Ser6366Ile = ex122m, p.Thr7382Pro = ex151m. Abbreviations: Tmod, tropomodulin; M1-M8, M163-M176, M177-M185 simple repeats; S1R1-S1R7, S22R1-S22R7, super repeats of seven simple repeats; Ser, Serine-rich domain, SH3, Src homology domain.

The successful isolation of the nebulin protein for the first time [33], and the generation of knockout mouse models [24,29,34] have helped elucidate the function of this giant molecule. Because of the enormous size of nebulin, functional studies of full-length nebulin are difficult. Hence, we opted for studying protein domains (super repeats) containing mutations known to cause NM (Figure 1). Mutations in all the selected super repeats have been reported to cause NM or distal myopathy (Table 1).

**Table 1:** **NM-causing *NEB *** **mutations included in the study**

**Fragment**	**Mutations in cDNA**^**a**^	**Altered protein site**	**Disease**	**Reference**
Neb ex53-57 (super repeat 9) in figures ex54m	c.7291G > A	p.Glu2431Lys	NM, mild form	Lehtokari *et al*. [18]
Neb ex53-57 (super repeat 9) in figures ex55del	c.7432 + 1916_7535 + 372del	p.Arg2478_Asp2512del	NM, severe, intermediate and typical forms	Lehtokari *et al*. [18]
Neb ex 77–81 (super repeat 14) in figures ex78del or ex78m	c.11770_11787del	p.Val3924_Asn3929del	NM, mild form	Lehtokari *et al*. [18]
Neb ex 119–125 (super repeat 18) in figures ex122m	c.19097G > T	p.Ser6366Ile	NM, typical form, distal myopathy	Lehtokari *et al*. [18]
Neb ex 146–153 (super repeat 22) in figures 151m	c.22144A > C	p.Thr7382Pro	NM, typical form, distal myopathy	Lehtokari *et al*. [18], Wallgren-Pettersson *et al*. [14]

NEB, Nebulin; NM, Nemaline myopathy.

^a^The site of the mutations is reported according to the coding sequence of *NEB* cDNA GenBank ID NM_001164507.1 and its translation, NP_001157979.1.

We produced four wild-type (WT) nebulin super repeats (283 to 347 aa long) and five corresponding mutants: three missense mutations (p.Glu2431Lys, p.Ser6366Ile, and p.Thr7382Pro) and two in-frame deletions (p.Arg2478_Asp2512del and p.Val3924_Asn3929del) (Table 1, Figure 1). The p.Arg2478_Asp2512del (2.5 kb deletion including exon 55) is a founder mutation in the Ashkenazi Jewish population. The missense mutations p.Ser6366Ile and p.Thr7382Pro are founder mutations in the Finnish population [14,18]. We performed F-actin and tropomyosin-binding experiments for the nebulin super repeats, using co-sedimentation and GST-pull-down assays in order to elucidate the pathogenetic mechanisms by which the mutations exert their effects.

## Methods

### RNA isolation and RT-PCR

Total RNA was isolated from human vastus lateralis (VL) muscles, using the RNeasy Fibrous Tissue Mini Kit (Qiagen, Venlo, The Netherlands). cDNA was synthesized from 2 μg of total RNA using the High-Capacity cDNA Reverse Transcription Kit (Applied Biosystems, Foster City, CA, USA); 2 μl of template were used per 20 μl PCR reaction. All PCR reagents were from Thermo Scientific (Waltham, MA, USA). The amplifications were performed using Phusion High-Fidelity DNA polymerase, and PCR products were cloned into pCRBluntII-TOPO (Invitrogen, Carlsbad, CA, USA). For all nebulin exon amplifications, after initial heating at 98°C for 1 minute, 30 cycles of denaturation at 98°C for 10 seconds, annealing at 59°C for 30 seconds, and extension at 72°C for 15 seconds were performed, followed by a final extension of 10 minutes at 72°C. The primers used for nebulin exon amplifications and *in vitro* mutagenesis are summarized in Table 2.

**Table 2:** **The oligonucleotide primers used for cloning the nebulin super repeats and for *in vitro *** **mutagenesis**

	**Primer name**	**5′-3′ Sequence**	**Experimental use**
1	Ex 54-F	CGC GAA TTC CAA GGC TAC CGA AAG CAA	cDNA amplification
2	Ex 54-R	CCG CTC GAG ATC GCT CTG GAG GTC ATA GGC	cDNA amplification
3	Ex 78-F2	TAC GGA TCC AAG TAC AAG GAA GGC TAC CG	cDNA amplification
4	Ex 78-R1	TTT CTC GAG TGC TTG GAT AAT GTC GTT TTG	cDNA amplification
5	Ex 122-F1	GCG GGA TCC GAG AAG CAG AAA GGT CAC TAC	cDNA amplification
6	Ex 122-R	CCG CTC GAG GAT GTT AAG CTT GCC AAC TCG	cDNA amplification
7	Ex 151-F	CGC GAA TTC AAA TTG GAA TAC AAC AAG GCC	cDNA amplification
8	Ex 151-R	CCG CTC GAG TTT GGC TGC CTG TGT GGC	cDNA amplification
9	Ex 54MUT-F	GATGGAGTCCCTTGGGTTCTTTAAAGGCAGAAAAGAAC	*In vitro* mutagenesis
10	Ex 54MUT-R	GTTCTTTTCTGCCTTTAAAGAACCCAAGGGACTCCATC	*In vitro* mutagenesis
11	Ex 78DEL-F	CATTACCGACACTCCGGAAATTGCCCTGACAATGAGCAAG	*In vitro* mutagenesis
12	Ex 78DEL-R	CTTGCTCATTGTCAGGGCAATTTCCGGAGTGTCGGTAATG	*In vitro* mutagenesis
13	Ex 122MUT-F	ACTGCTGTTCAGAGTGGCATTAATGCCATTGAGGTAAAATATAA	*In vitro* mutagenesis
14	Ex 122MUT R	TTATATTTTACCTCAATGGCATTAATGCCACTCTGAACAGCAGT	*In vitro* mutagenesis
15	Ex 151MUT-F	TTCACGTCAAGGAAGTGCCCAAGCATGTCAGTGAT	*In vitro* mutagenesis
16	Ex 151MUT R	ATCACTGACATGCTTGGGCACTTCCTTGACGTGAA	*In vitro* mutagenesis
17	Ex 78DEL-F	CATTACCGACACTCCGGAAATTGCCCTGACAATGAGCAAG	*In vitro* mutagenesis
18	Ex 78DEL-R	CTTGCTCATTGTCAGGGCAATTTCCGGAGTGTCGGTAATG	*In vitro* mutagenesis

### Ethics approval

The project as a whole was approved by the ethics committee of Children’s Hospital, University of Helsinki, Helsinki, Finland. The VL muscle was obtained from an amputated leg at Tampere University Hospital, and written informed consent for tissue sampling was given by the patient. Ethical approval for this sampling was given by the ethics committee of Tampere University Hospital, Tampere, Finland.

### *In vitro* mutagenesis and sequencing

QuickChange site-directed mutagenesis kit (Stratagene, La Jolla, CA, USA) was used to introduce site-specific point mutations or deletions into *NEB* cDNA fragments. PCR reactions were performed using a supercoiled, double-stranded DNA as template, with two synthetic oligonucleotide primers containing the chosen mutation. Cycling conditions were as recommended in the manual, and the primer details are given in Table 2. The purified products were sequenced using BigDye sequencing chemistry (version 3.1_ and an ABI 3730 DNA Analyzer (Applied Biosystems, Foster City, CA, USA). The sequences were analyzed using the Sequencher 4.1 software (Gene Codes Corporation, Ann Arbor, MI, USA).

### Construction of vectors for the expression of nebulin super repeats

Plasmid vectors for the expression of human nebulin super repeats 14 and 18 for the analysis of exon 78 and exon 122 were constructed by cloning digested and purified PCR products into the *Bam*HI/*Xho*I restriction sites of the pGEX4T-1 expression vector. Plasmid vectors for the expression of super repeats 9 and 22 for the study of exon 54 and exon 151 were constructed by cloning digested and purified PCR products into the *Eco*RI/*Xho*I restriction sites of the pGEX4T-1 expression vector.

### Protein production in *Escherichia coli*

GST-nebulin fusion proteins were expressed from the pGEX-4 T vectors in the *E. coli* strain BL21 (DE3) (Invitrogen). The proteins were expressed by selecting a single colony and culturing in 5 ml LB supplemented with ampicillin 100 μg/ml. After growing the *E. coli* to absorbance of 0.5 to 0.8 at 600 nm, the cells were induced with 0.5 mM isopropyl-β-D-thiogalactopyranoside (IPTG) for 3 hours at 250 rpm and 27.8°C. Harvesting of cells and batch-binding protein purifications were performed as described in the manufacturer’s manual (Protino® Glutathione Agarose 4B; Macherey-Nagel, Düren, Germany).

### Production of wild-type and aberrant α-tropomyosin and β-tropomyosin

The α-tropomyosin and β-tropomyosin were expressed in a baculovirus/*Sf9* system (Invitrogen) and purified according to previously described protocols [35,36]. The insect cells were grown at 27°C in supplemented Grace’s Insect Medium (Invitrogen) containing 10% fetal bovine serum (Gibco) and 1% penicillin/streptomycin (Gibco).

### Actin binding

Actin binding assays were performed using the Actin Binding Protein Biochem Kit (Cytoskeleton, Denver, CO, USA). Nebulin super repeats (10 μg) were allowed to bind to F-actin (40 μg) for 30 minutes at room temperature. The samples were run in a Beckman Coulter Optima MAX Ultracentrifuge at 60,000 rpm for 1.5 hours. The pellet and supernatant fractions were separated and analyzed by 12% SDS-PAGE electrophoresis, and the proteins were stained with Coomassie Blue. The pellet and supernatant bands were quantified from three experiments done in duplicates using the ImageJ Program (National Institutes of Health, Bethesda, MD, USA).

### GST-pull-down assay

GST-containing WT and mutant nebulin super repeats (8 μg) bound to beads were mixed with baculovirus-produced and purified WT α-tropomyosin, WT β-tropomyosin, and mutant β-tropomyosin (40 μg) in 400 μl PBS in a shaker at 4°C for 45 minutes. Samples were centrifuged at 2000 rpm (500 x g) for 5 minutes. The supernatant was removed, and 400 μl fresh 1xPBS was added. Samples were washed five times with centrifugation at 1500 to 2000 rpm (300 to 500 x g) for 5 minutes. The pellets were dissolved in 20 μl Laemmli sample buffer, and analyzed by 12% SDS-PAGE electrophoresis. The proteins were stained with Coomassie Blue. Bands of pelleted protein were quantified using the ImageJ Program from three experiments performed in duplicate.

We also tested WT nebulin super repeat binding affinity to WT α-tropomyosin and β-tropomyosin at different concentrations using GST pull-down. Nebulin super repeats (8 μg) were mixed with WT α-tropomyosin and WT β-tropomyosin (7.5, 15, 30, and 60 μg).

### Statistical analysis

The statistical significance of the results was calculated using the Mann–Whitney test when comparing two groups, and the Kruskal–Wallis test when comparing three or more groups.

## Results

We produced four WT 283 to 347 aa long nebulin super repeats: S9 (347 aa), S14 (347 aa), S18 (283 aa), and S22 (284 aa), and five corresponding mutants (Figure 1). The individual simple repeats (seven in each super repeat) were of slightly different lengths in different super repeats, hence the size difference. We compared the binding affinities of WT protein domains with the corresponding mutants.

**Figure 2:**
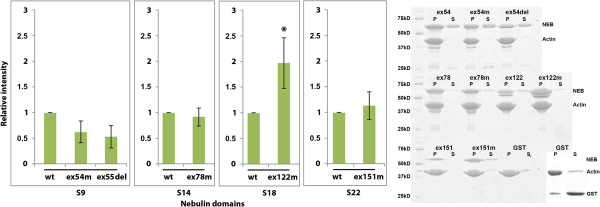
**Mutations affect the binding of nebulin to F-actin.** Nebulin protein domains were incubated with F-actin and centrifuged. Pellet and supernatant fractions were separated, run on SDS-PAGE gels, and stained with Coomassie Blue. The relative abundance of nebulin protein in the pellet was quantified from three independent experiments. The mean value and standard deviations from three experiments are shown in the bar charts to the left, and gel pictures of representative experiments are shown on the right. Nebulin domains containing the mutation p.Ser6366Ile (ex122m) showed significantly strengthened actin affinity (*P* = 0.048). *P* values were calculated using the Kruskal–Wallis test when comparing three groups (S9) and the Mann–Whitney test when comparing two groups (S14, S18, S22). Asterisks indicate significant differences compared with the wild-type (WT) protein.

Nebulin super repeat 9, containing the p.Glu2431Lys (exon 54, c.7291G > A) mutation (which is close to a putative tropomyosin-binding site), and nebulin super repeat 9, lacking 35 aa due to deletion of the entire exon 55 (p.Arg2478_Asp2512del, c.7431 + 1916_7536 + 372del), showed weaker affinity for F-actin compared with the WT fragments, but the difference was not confirmed to be statistically significant using the Kruskal–Wallis test (Figure 2). Super repeat 18, containing the p.Ser6366Ile (exon 122, c.19097G > T) mutation at an actin-binding site, showed stronger actin affinity (*P* = 0.048, Figure 2). Super repeat 14, containing an in-frame deletion of six aa (exon 78, p.Val3924_Asn3929del), and super repeat 22, containing the p.Thr7382Pro (exon 151, c.22144A > C) mutation, showed similar affinity for actin as the WT fragments (Figure 2).

**Figure 3:**
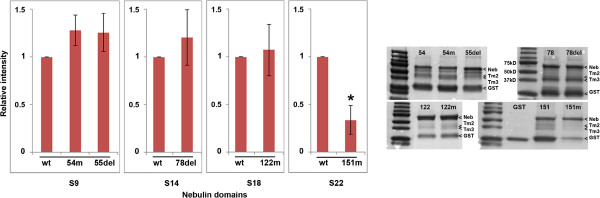
**Nebulin mutations affect binding to tropomyosin.** Purified GST-nebulin domains bound to beads were incubated with purified α-tropomyosin and β-tropomyosin, then beads were washed and the bound proteins run in SDS-PAGE gels, and stained with Coomassie Blue. The relative intensity of bound α-tropomyosin and β-tropomyosin was quantified from three independent experiments. The mean value and standard deviations from three experiments are shown in the bar charts to the left, and gel pictures of representative experiments are shown on the right. Nebulin domains containing the p.Thr7382Pro (ex151m) mutation showed significantly weakened affinity for tropomyosin than wild-type (WT) proteins (*P* = 0.039). *P* values were calculated using the Kruskal–Wallis test when comparing three groups (S9) and the Mann–Whitney test when comparing two groups (S14, S18, S22). Asterisks indicate significant differences compared with the WT protein.

**Figure 4:**
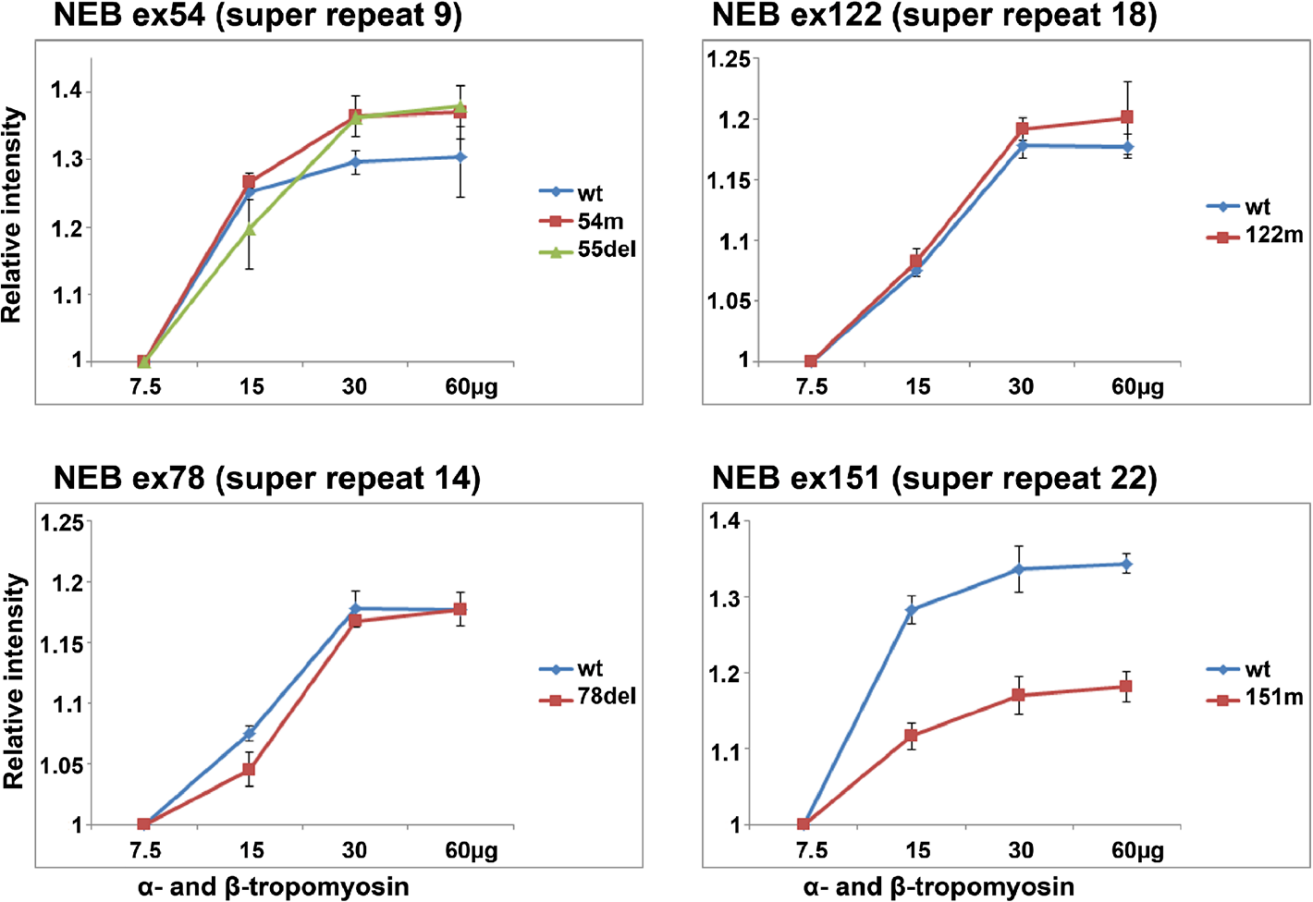
**Tropomyosin-nebulin binding curves.** The affinity of wild-type (WT) and mutant nebulin domains to α-tropomyosin and β-tropomyosin is shown in binding curves. The relative quantities were calculated from three independent experiments.

**Figure 5:**
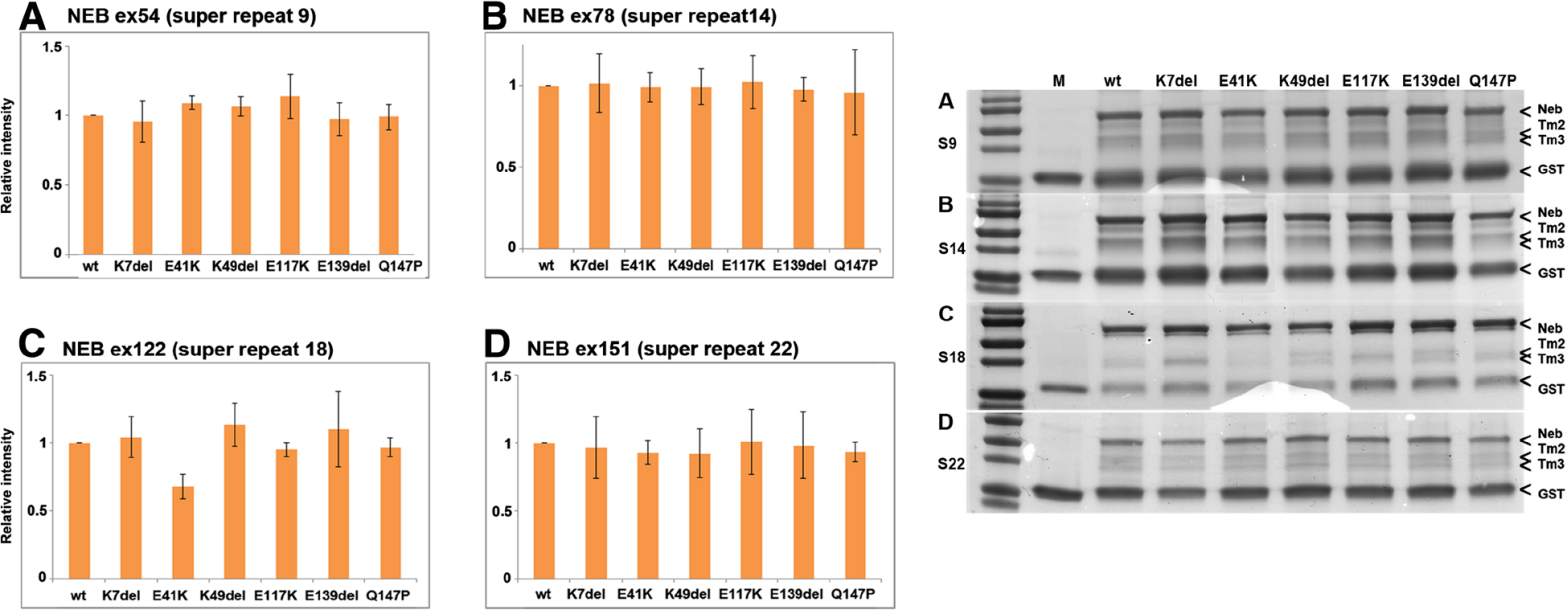
**Nebulin wild-type (WT) super repeat domain affinity for mutant tropomyosins.** Purified GST-nebulin domains bound to beads were incubated with purified α-tropomyosin and β-tropomyosin, then beads were washed, and bound proteins were run in SDS-PAGE gels and stained with Coomassie Blue. The relative quantities of bound α-tropomyosin and β-tropomyosin were calculated from three independent experiments. Six β–tropomyosin mutants (p.K7del, p.E41K, p.K49del, p.E117K, p.E139del, and p.Q147P) were included in the study. The mean values and standard deviations from three experiments are shown in the bar chart to the left, and gel pictures of representative experiments are shown on the right. **(A)** Binding affinities of the β–tropomyosin mutants for nebulin super repeat S9. **(B)** Binding affinities of the β–tropomyosin mutants for nebulin super repeat S14. **(C)** Binding affinities of the β–tropomyosin mutants for nebulin super repeat S18. **(D)** Binding affinities of the β–tropomyosin mutants for nebulin super repeat S22. No statistically significant differences in binding affinities were found. Abbreviations: Neb, nebulin; Tm2, β-tropomyosin; Tm3, α-tropomyosin; GST, glutathione-S-transferase.

We also performed tropomyosin-binding experiments for the super repeats using GST pull-down assays (Figure 3). Some of the produced nebulin domains showed degraded fragments, but these were larger in size than tropomyosin (Figure 1B). Super repeat 9, containing the p.Glu2431Lys mutation, showed stronger affinity for tropomyosin but this was not confirmed to be statistically significant. Super repeat 9, containing the in-frame deletion of exon 55 (p.Arg2478_Asp2512del), and super repeat 14, containing the in-frame deletion p.Val3924_Asn3929del, showed slightly, but not statistically significant, stronger affinity for tropomyosin. Super repeat 18, containing the p.Ser6366Ile mutation, showed similar affinity for tropomyosin as WT fragments. The nebulin exon 151, containing super repeat 22 with the missense mutation p.Thr7382Pro, showed significantly weaker affinity for tropomyosin compared with the WT protein fragment (*P* = 0.039). Tropomyosin affinities for each nebulin super repeat are shown as binding curves (Figure 4).We also tested the affinity of WT nebulin super repeats for WT and six β–tropomyosin mutants (p.Lys7del, p.Glu41Lys, p.Lys49del, p.Glu117Lys, p.Glu139del, and p.Gln147Pro) using GST-pull-down assays. Nebulin super repeat 18 containing the WT exon 122 showed slightly weaker affinity for the β-tropomyosin p.Glu41Lys mutant, but this was not statistically significant using the Kruskal–Wallis test. The other mutant tropomyosins did not show significant differences in binding affinity for WT nebulin compared with WT tropomyosin (Figure 5).

## Discussion

The WLKGIGW motif in nebulin has been proposed to serve as a tropomyosin-binding site [21,23]. Performing GST-pull-down assays for four WT nebulin super repeats (9, 14, 18, and 22) and WT α- and β-tropomyosins, we showed that all four nebulin super repeats bound to tropomyosin with high affinity. This is the first direct evidence that there is a tropomyosin-binding motif in these super repeats of nebulin (Figure 4, Figure 5). Chitose and co-workers [33] used far-western blotting and whole nebulin from rabbit skeletal muscle to test tropomyosin binding. They were not able to confirm any interaction between nebulin and tropomyosin. This could be due to lower quantities of protein or differences in testing methods. Moreover, there could be an advantage in using smaller protein domains that can adopt the correct α-helical conformations.

Nebulin knockout mouse models and analyses of single muscle fibers from patients with NM caused by mutations in *NEB* have provided some insights into the pathogenesis of NM [24,34,37-41], but *in vitro* functional studies of *NEB* mutations have not been performed previously, to our knowledge. Furthermore, the functional effects of *NEB* missense mutations have not been addressed to date.

Recent studies have shown that patients with NM caused by mutations in *NEB* may have markedly lower levels of nebulin protein in their muscles than healthy individuals, leading to lower calcium sensitivity of force generation [37,39,41]. A lower abundance of nebulin has been associated with the in-frame deletion of exon 55 (p.Arg2478_Asp2512del) included in the present study, as well as with frameshift and splice-site mutations in *NEB*[39,41]. It has been suggested that the vulnerability of mutant nebulin to proteolysis is due to a mismatch between nebulin and its actin-binding sites [38]. The results of our nebulin-actin binding studies support this suggestion, as the super repeat lacking 35 aa encoded by exon 55 (S9) showed weakened actin affinity, although the difference was not statistically significant. The 35 aa deletion does not span the tropomyosin-binding site in super repeat 9 (Figure 1A), and super repeat S9 showed slightly, but not statistically significantly, stronger binding to tropomyosin (Figure 3). The effect may be more pronounced *in vivo,* when the tropomyosin-binding site periodicity of 235 to 240 aa is disrupted by the deletion, and the head-to-tail binding of tropomyosin dimers to the thin filament might thus be impaired [18].

Interestingly, nebulin super repeat S9 containing the p.Glu2431Lys mutation, which is close to a tropomyosin-binding site (Figure 1A) showed weakened actin affinity (Figure 2), but also stronger affinity for tropomyosin (Figure 3). These differences were not shown to be statistically significant. This mutation was identified in a patient with a mild form of NM, who is compound heterozygous for the p.Glu2431Lys mutation and a frameshift mutation in exon 55 (Lehtokari *et al*., manuscript submitted). Stronger tropomyosin affinity may impair the movement of nebulin in concert with tropomyosin on the actin filament during muscle contraction. Incomplete movement of tropomyosin on the actin filament, resulting in disrupted myosin cross-bridge cycling kinetics and subsequent muscle weakness, has been described in muscle fibers from a patient with NM who was compound heterozygous for two splice-site mutations in *NEB*. The mutations resulted in skipping of exons 3 and 22. In that patient, the abundance of nebulin in muscle was only slightly lower than normal, and the calcium sensitivity of force production was maintained [40].

The missense mutations p.Ser6366Ile in super repeat S18 and p.Thr7382Pro in S22 are founder mutations in the Finnish population, and had been discovered in compound heterozygous form, together with a truncating mutation (frameshift or nonsense), to cause NM, and in a homozygous form to cause distal myopathy without nemaline bodies [14,18]. Interestingly, the p.Ser6366Ile mutation significantly strengthened the actin affinity of super repeat S18 (Figure 2). The strengthened actin affinity may have an impact on actin-myosin interaction during muscle contraction, considering that one nebulin-binding site on actin is in close proximity to the strong binding site for myosin on actin during muscle contraction, and that this site is blocked by tropomyosin in relaxed muscle [32]. The p.Thr7382Pro mutation did not affect actin affinity, but significantly weakened the tropomyosin affinity of super repeat S22 (Figure 3), although the mutated amino acid is much closer to an actin-binding site than to the tropomyosin-binding site (Figure 1A). Super repeat S22 is the last super repeat before the C-terminal simple repeat region of nebulin, and it also contains the last predicted tropomyosin-binding site in nebulin [42].

The in-frame deletion of six aa (p.Val3924_Asn3929del) in super repeat S14 had no effect on actin or tropomyosin binding. This deletion does not reside in a known binding site. A few small (1 to 5 aa) in-frame deletions and insertions in *NEB* are listed in the Exome Variant Server (EVS) [43], compiling exome sequencing data of healthy individuals, as well as individuals with hypertension, and heart and lung disease. To our knowledge, no patients with skeletal muscle disease are included in the EVS study cohorts. Of note, some individuals in the EVS are homozygous for the in-frame deletions, indicating that at least some small in-frame deletions in *NEB* are non-pathogenic. The p.Val3924_Asn3929del in-frame deletion in our study (not present in the EVS) is the only small in-frame deletion we detected in our large series of patients with NM. The patient is compound heterozygous for p.Val3924_Asn3929del and a large (approximately 30 kb) duplication in *NEB* (unpublished results). The pathogenicity of both mutations remains to be established.

We also tested the affinity of six β–tropomyosin mutants (p.Lys7del, p.Glu41Lys, p.Lys49del, p.Glu117Lys, p.Glu139del, and p.Gln147Pro) for WT nebulin super repeats using the GST pull-down assay. The tropomyosin p.Glu41Lys mutant showed a slightly weakened affinity for nebulin super repeat S18, which was not statistically significant, but not for the other super repeats. The p.Glu41Lys substitution is in the non-actin-binding β-zone of β-tropomyosin and has been shown to cause low Ca^2+^ sensitivity by *in vitro* motility assays [35]. The other tropomyosin mutations, except p.Glu117Lys, are in or close to the tropomyosin-actin-binding site at the α-zones of β-tropomyosin [44,45]. These mutants did not show significant changes in binding affinity for WT nebulin super repeats compared with WT proteins (Figure 5).

## Conclusions

Our results demonstrate actin-nebulin and tropomyosin-nebulin interactions *in vitro*, and show that mutations in nebulin and tropomyosin can alter these interactions. Both actin and tropomyosin-binding affinity was affected by nebulin mutations. This suggests that abnormal interaction between aberrant thin-filament proteins is a pathogenetic mechanism in NM and related disorders.

## Abbreviations

ACTA1: alpha-actin 1; EVS: Exome Variant Server; GST: Glutathione-S-transferase; NEB: Nebulin; NM: Nemaline myopathy; PBS: Phosphate buffered saline; SDS-PAGE: Sodium dodecyl sulfate polyacrylamide gel electrophoresis; Tm: Tropomyosin; VL: Vastus lateralis; WT: Wild-type.

## Competing interests

The authors declare that they have no competing interests.

## Authors’ contributions

MH performed the cloning and site-directed mutagenesis. Nebulin protein fragments were produced by MH and MM. Tropomyosins were produced and purified by EL and KJN. MH performed the nebulin-actin binding experiments together with EL. MM studied WT and mutant nebulin binding to WT tropomyosins. MM did the experiments on WT nebulin binding to WT and mutant tropomyosins. KP and JL produced the figure on nebulin super repeats. MM, MH, KP, MG, and CWP planned the study and wrote the article. All authors read and approved the final manuscript.
